# Context-Dependent Strategies for Enhanced Genome Editing of Genodermatoses

**DOI:** 10.3390/cells9010112

**Published:** 2020-01-02

**Authors:** Oliver Patrick March, Thomas Kocher, Ulrich Koller

**Affiliations:** EB House Austria, Research Program for Molecular Therapy of Genodermatoses, Department of Dermatology and Allergology, University Hospital of the Paracelsus Medical University Salzburg, 5020 Salzburg, Austria; opmarch92@gmail.com (O.P.M.); t.kocher@salk.at (T.K.)

**Keywords:** genome editing, genodermatoses, designer nucleases, DNA repair pathways, epidermolysis bullosa, keratinopathies

## Abstract

The skin provides direct protection to the human body from assault by the harsh external environment. The crucial function of this organ is significantly disrupted in genodermatoses patients. Genodermatoses comprise a heterogeneous group of largely monogenetic skin disorders, typically involving mutations in genes encoding structural proteins. Therapeutic options for this debilitating group of diseases, including epidermolysis bullosa, primarily consist of wound management. Genome editing approaches co-opt double-strand break repair pathways to introduce desired sequence alterations at specific loci. Rapid advances in genome editing technologies have the potential to propel novel genetic therapies into the clinic. However, the associated phenotypes of many mutations may be treated via several genome editing strategies. Therefore, for potential clinical applications, implementation of efficient approaches based upon mutation, gene and disease context is necessary. Here, we describe current genome editing approaches for the treatment of genodermatoses, along with a discussion of the optimal strategy for each genetic context, in order to achieve enhanced genome editing approaches.

## 1. Introduction

### 1.1. The Anatomy of the Skin

**Figure 1:**
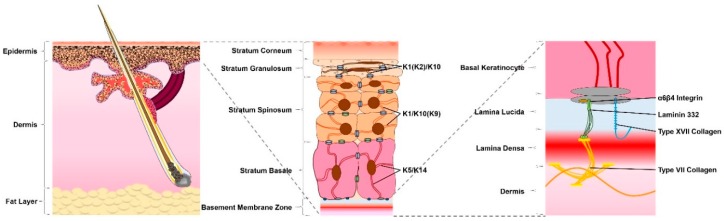
The anatomy of the skin. The skin consists of three layers: fat, dermis and epidermis. The epidermis is typically composed of four layers, situated above the basement membrane. From inner to outer, these are: the stratum basale, stratum spinosum, stratum granulosum and stratum corneum—associated with different expression profiles with respect to keratins and intracellular adhesions. Keratins 5 and 14 (K5/K14) are expressed in basal cells. Suprabasal cells demonstrate a downregulation of K5/K14 in favor of K1/K10 in normal interfollicular epidermis and K1/K9 in the palms and soles. Cells of the stratum granulosum additionally express K2. The basal keratinocytes of the stratum basale connect the epidermis to the basement membrane zone, comprised of the lamina lucida and lamina densa, which provides connection between the epidermis and the underlying dermis. Structural proteins within the basement membrane zone, such as α6β4 integrin, laminin 332, type XVII collagen and type VII collagen, are crucial for the integrity and stability of the skin.

The skin is the largest organ of the human body, consisting of three main layers, with differing cellular localization, organization, expression and function (Figure 1) [1]. The deepest of these is the underlying subcutaneous fat layer, which contacts skeletal muscle. Above the subcutaneous fat layer, lies the dermis—an elastic connective tissue, separated from the outermost skin layer by the basement membrane [2]. Also termed the dermal–epidermal junction (DEJ), this complex structure consists of a variety of proteins, such as integrins, collagen fibers and laminins [3]. The basement membrane is essential for adhesion and signaling between the dermis and the much thinner, outermost layer of the skin—the epidermis [4]. This is a multi-layered, stratified squamous epithelium, largely composed of keratinocytes and capable of continuous regeneration [5]. The intermediate filaments (IFs) of these cells are formed from keratins (Ks) and provide the tensile strength of the skin for immediate protection from the environment [6]. Weakening of the skin through disease or wounding disrupts these functions, leaving the body vulnerable to assault and further damage.

### 1.2. Gene Therapies for Genodermatoses

Genodermatoses comprise a heterogeneous group of largely monogenetic skin disorders [7]. Therapeutic genodermatoses targets largely comprise structural epidermal genes such as the keratins and filaggrin leading to ichthyoses, laminins, integrins and collagens leading to blistering disorders and genes encoding components of DNA repair pathways [8]. In addition to the diverse group of genodermatoses-associated genes, associated mutations demonstrate significant heterogeneity, resulting in a variety of phenotypic severities and outcomes.

To date, treatment options have in general been limited to symptom relief, although the regenerative capacity and accessibility of the skin make it an attractive target for genetic therapies. Auspicious treatment outcomes have been achieved with viral ex vivo gene addition approaches [9,10,11]. However, several limitations remain. In particular, these approaches are preferentially suitable for recessive disease forms involving small genes.

Recent and rapidly increasing advances in the field of genome editing provide alternatives to gene addition, circumventing many issues with this older technology. A variety of genome editing technologies have been employed in the development of therapeutic approaches for skin diseases, each associated with unique benefits and shortfalls [12]. However, aside from the ease and cost of generation and use, all genome editing technologies rely on the formation of specific double-strand breaks (DSBs) and their resolution via DSB repair pathways. It is therefore the targeting strategy, nature, and context of the DSB that determine the efficiency and outcome of genome editing approaches, rather than nuclease choice.

### 1.3. DNA Repair Pathways

**Figure 2:**
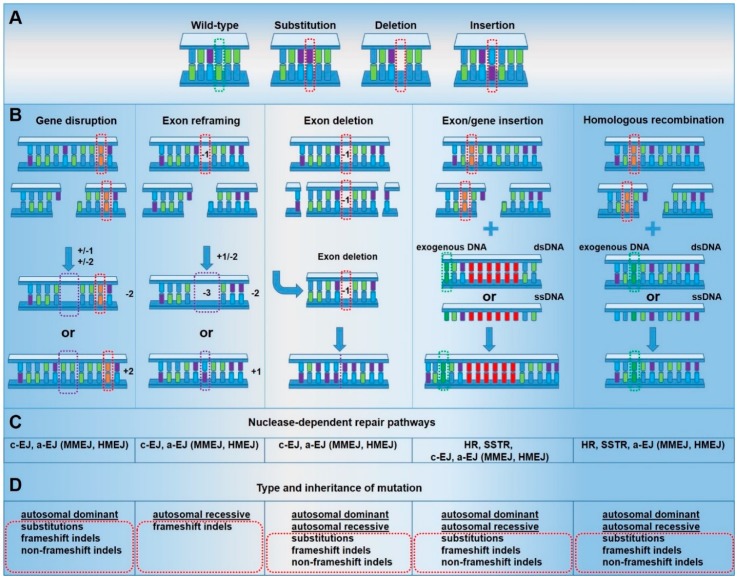
Context-dependent gene repair strategies. Genome editing technologies rely on the formation of specific double-strand breaks (DSBs) within a gene and their resolution via DSB repair pathways. The targeting strategy, nature, and context of the DSB determine the repair outcome of the different genome editing approaches. Therefore, the choice of an efficient targeting strategy for genome editing of genodermatoses is highly dependent on the type and inheritance of the mutation. (**A**) Types of mutations. (**B**) Gene repair strategies. (**C**) Nuclease-dependent DNA repair pathways. (**D**) Type and inheritance of mutation, which can be targeted by the respective gene repair strategy. (Orange bars and red dotted rectangles indicate the mutation site; dark green bars and dotted rectangles indicate wild-type (wt) sequence; purple dotted rectangles indicate wanted repair outcomes; red bars indicate exon/gene insertion.)

Following the generation of a DSB, cellular DNA repair pathways are activated in a cell cycle- and context-specific manner. Currently, multiple pathways have been implicated in genome editing-induced DSBs [13] (Figure 2). The largest proportion of DSBs is repaired via classical end joining (classical EJ), which is rapidly activated [14,15] throughout all phases of the cell cycle [16]. This pathway involves direct ligation of the two DNA ends in a frequently perfect manner [17]. However, it is also associated with the introduction of small insertions and deletions [14]. Classical EJ-based repair can therefore be co-opted for gene disruption, gene reframing and exon deletion, the mutagenic efficiency of which may be limited by perfect DSB repair outcomes [16]. If classical EJ fails to repair the DSB, then 5′ to 3′ resectioning of the DNA ends takes place, to generate 3′ single-stranded overhangs, suitable for slower [18] and highly mutagenic [19] alternative end joining (alternative EJ) pathways [20,21], such as microhomology-mediated end-joining (MMEJ) and theta-mediated end-joining (TMEJ) [22]. Active throughout gap-1 (G1) / early synthesis (S) phases of the cell cycle, alternative EJ pathways utilize small areas of homology on 3′ overhangs to ligate the two DNA ends together [14]. This necessarily leads to larger deletions in the case of MMEJ [19,21] and insertions in the case of TMEJ [23,24], which can be co-opted for more efficient but imprecise gene disruption, gene reframing, exon deletion and, in the presence of a homologous DNA template, exon/gene insertion.

Following the failure of alternative EJ pathways, and in the presence of a double-stranded DNA template bearing homology to the area of the DSB, homologous recombination (HR) pathways are activated [25]. HR occurs exclusively during the S and G2 phases of the cell cycle in proliferating cells, when intact sister chromatids are available for use as repair templates [22], reaching peak activity in mid-S phase [26]. HR utilizes previously generated 3′ single-stranded overhangs to base pair with the complementary template [22]. Subsequently, DNA complementary to the template is synthesized prior to ligation of the resulting sequence to the free DNA end [27,28,29]. This repair pathway is typically associated with perfect DSB repair outcomes, although mutagenesis occurs at higher frequencies than standard DNA replication [30]. Distinct from HR, single-strand template repair (SSTR) involves the annealing of a single-stranded DNA template with resected 3′ overhangs at the DSB. However, the mechanism of this pathway requires further elucidation [25]. HR and SSTR can be co-opted for the introduction of small and large sequence variations at the nuclease target site, including exon/gene insertions. However, in gene editing experiments, the frequencies of HR and SSTR are often lower due to the dominance of other pathways, such as classical EJ [16]. Therefore, these approaches are frequently implemented in strategies where gene disruption and reframing are unsuitable.

### 1.4. Genome Editing Strategies for Genodermatoses

Efficient, precise phenotypic correction of genodermatoses via genome editing is critically reliant on the prior definition of an effective, defined targeting strategy. However, the associated phenotypes of many mutations may be treated through the implementation of several approaches. The simplest and most efficient genome editing strategies employ EJ-based DSB repair for gene disruption [31,32,33,34] and gene reframing [35,36,37]. As EJ-based strategies do not require a DNA donor template, potentially detrimental integrations and DNA-associated toxicity are avoided. Recently, exon deletion approaches have been developed for highly efficient, homogenous gene reframing strategies that can be used to treat disease hotspots in non-patient-specific manner [38,39]. However, as these approaches rely on the introduction of insertions and deletions within target loci, they are unsuitable for many mutations and diseases. It is primarily for this reason that the earliest applications of genome editing in genodermatoses typically involved exon/gene insertions [35,40,41,42,43,44,45,46]. These strategies primarily involved the HR-based replacement of pathogenic exons with a non-pathogenic sequence encoding intronic selection markers, enabling simpler isolation of corrected cells, from early-stage and therefore inefficient approaches. However, with the advent of optimized HR strategies, selection-free approaches are favored, featuring minimal sequence divergence from the wild-type. These less artificial systems avoid the introduction of non-human sequences into the genome and therefore hold greater clinical applicability.

An efficient targeting strategy for genome editing of genodermatoses takes the context of the gene, mutation and disease form into account, with the most efficient available strategy typically selected (see summary in Table 1).

## 2. Genome Editing Applications for Genodermatoses

### 2.1. Gene Disruption

Genome editing-mediated disruption of pathogenic alleles is an effective therapeutic strategy for the treatment of dominant-negative skin diseases [32,34]. Gene disruption can be achieved via the generation of a single DSB, followed by error-prone EJ-based repair, to introduce indels into the target gene. Nuclease-induced indels frequently lead to frameshifts within the target allele, which will in turn induce premature termination codons (PTCs). In many cases, induction of PTCs leads to nonsense-mediated mRNA decay (NMD) [53]. As approximately two thirds of all nuclease-induced indels lead to PTC induction, this approach can result in very high gene disruption efficiencies, without extensive screening [34]. Within genodermatoses, gene disruption is only suitable for the phenotypic correction of heterozygous autosomal dominant disease forms, whereby knockout of a single mutant allele would provide phenotypic alleviation. Frameshift-inducing indel mutations necessarily comprise the only feasible targets of exon reframing approaches. However, as PTCs can lead to protein truncation or be subject to read-through, full prior characterization of PTC targets is necessary [34].

The majority of genome editing-mediated gene disruption approaches in genodermatoses have targeted keratinopathies. These are a group of disorders including epidermolysis bullosa simplex (EBS), epidermolytic ichthyosis (EI) and pachyonychia congenita (PC), largely caused by dominant-negative mutations in keratins expressed within specific epidermal layers of the skin [54]. Keratins form the heteropolymeric intermediate filament (IF) network of epidermal keratinocytes [55]. Dominant keratin mutations, which are predominantly missense mutations, incorporate into this network. The structural imperfections within mutant keratins resulting in cytoskeletal fragility of epidermal cells [56,57,58,59]. As heterozygous recessive mutations in keratins do not lead to disease [60,61], gene disruption of heterozygous dominant-negative mutations comprise efficient strategies for phenotypic alleviation of many keratinopathic patients.

In 2017, Aushev et al. [32] presented an unbiased targeting strategy to disrupt mutant *KRT5* alleles via EJ-based genome editing, aiming to leave the wild-type allele intact. Without the use of selection markers, a gene editing protocol based on screening and isolation of edited keratinocytes was developed and proved to be functional in immortalized EBS patient keratinocytes [32]. The authors used a non-allele-specific targeting strategy potentially applicable for many EBS patients with *KRT5* mutations. An allele-specific inactivation would be difficult to implement for clinical translation as, for each dominant-negative mutation, individual nucleases would require generation and validation in vitro. Further, only a subset of nuclease-induced indels lead to PTC induction that reliably results in NMD of the edited transcript. Some PTCs in keratins lead to read-through or expression of truncated protein products with dominant-negative activity. Inactivation of mutant *KRT5* alleles resulted in the elimination of any IF abnormalities associated with EBS keratinocytes [32].

Similar to this study, March et al. [34] recently exploited TALENs to disrupt the *KRT10* gene, in which dominant mutations cause epidermolytic ichthyosis (EI). The applicability of this approach was validated in immortalized and primary EI keratinocytes. To obtain a complete absence of mutant *KRT10* expression upon TALEN treatment, without translation of any truncated protein products, the *KRT10* gene was disrupted upstream of a PTC described to result in NMD of *KRT10* transcripts [60]. Gene disruption was also demonstrated for a previously uncharacterized PTC, induced via an alternative frameshift. NMD of *KRT10* transcripts via these two PTCs was demonstrated, representing an efficient strategy for eliminating mutant keratin 10 (K10) from EI patient cells. Without selection, an on-target gene editing efficiency of 56.8% was obtained in immortalized keratinocytes. Genome editing efficiencies of over 20% were subsequently described in treated primary EI keratinocytes. Western blot analysis of treated keratinocytes revealed a normalization of K10 expression in a gene-edited isolated single cell keratinocyte clone and the absence of any truncated keratins. This indicated full degradation of edited mutant *KRT10* mRNA [34].

Luan et al. [33] showed first in vivo data for nuclease-mediated knockout in skin cells in 2018. Correction of a dominant-negative mutation within *KRT9,* causing epidermolytic palmoplantar keratoderma (EPPK), was achieved using a mutation-specific CRISPR/Cas9-based strategy. The Cas9 nuclease and respective single guide RNA (sgRNA) were delivered via lentiviral vector into the forepaws of a transgenic mouse carrying a heterozygous indel mutation within *KRT9*. Following three injections, K9 expression was decreased by approximately 14.6%. The authors also assumed a visible restoration of the abnormal epidermal differentiation and proliferation [33].

Besides keratins, CRISPR/Cas9-mediated gene disruption was successfully applied in vitro and in vivo to correct dominant dystrophic epidermolysis bullosa (DDEB). Shinkuma et al. [31] developed an allele-specific gene editing approach in order to disrupt dominant-negative *COL7A1* alleles harboring a 15nt deletion. This mutation interferes with the formation of the collagen triple helix in a dominant fashion. Mutation-specific nucleases were generated and transfected into induced pluripotent stem cells (iPSCs) derived from primary patient fibroblasts. However, the resulting genome editing-induced indels led to PTCs not associated with NMD. Truncated C7 protein products were detectable in iPSCs differentiated into fibroblasts and keratinocytes, after *COL7A1* editing [31]. This underlines the importance of prior characterization of target PTCs in gene disruption approaches.

Gene disruption offers a simple, efficient strategy for the phenotypic correction of many dominant disorders. This is particularly true with the recent advent of precise CRISPR/Cas9 targeting [62]. Targeting of pathogenic indels with nucleases that generate highly homogenous DSB repair outcomes could lead to highly efficient approaches. However, in the absence of allele-specific strategies, single cell expansion and screening will be required to isolate cells with the mutant allele alone disrupted [32,34]. In vivo application of highly precise molecules is likely unfeasible due to this requirement. However, this strategy can be exploited to knockout dominant alleles with mutations at distinct locations via a single nuclease, providing a treatment option for almost all patients [34].

Keratinopathies comprise the majority of dominant genodermatoses and thus represent the most suitable disease group for gene disruption strategies. Nevertheless, many other genodermatoses are associated with recessively inherited diseases, and are therefore unsuitable. However, for the phenotypic correction of DDEB, targeting of an effective NMD-inducing PTC in *COL7A1* should be also explored.

### 2.2. Exon Reframing

Genome editing-mediated reading frame restoration of pathogenic alleles is an effective therapeutic strategy for Duchenne muscular dystrophy [63,64,65] and recessive DEB (RDEB) [35,36,37]. Exon reframing can be achieved in an identical manner to gene disruption approaches, as approximately one third of all nuclease-induced indels lead to codon restoration of frameshift mutations. As with gene disruption approaches, this approach can result in very high correction efficiencies, without extensive screening [37]. Exon reframing is mainly suitable for the correction of recessive disease forms, whereby rescue of gene expression would provide significant phenotypic alleviation. Frameshift-inducing indel mutations necessarily comprise the only feasible targets of exon reframing approaches. Additionally, only gene targets that are amenable to amino acid divergence from wild-type should be considered. As structural alterations resulting from altered amino acids might affect protein functionality, full characterization of protein products is necessary [37].

All exon reframing approaches for genodermatoses to date have targeted *COL7A1* [35,36,37]. This is largely due to the phenotypic severity of RDEB-causing frameshift mutations and the proven amenability of *COL7A1* to truncation and reading frame restoration, within the collagenous domains [66]. The contrastingly reduced severity of dominant-negative forms of the disease [59] indicates that, at minimum, expression of aberrant protein variants will provide therapeutic alleviation. However, maintenance of glycine residues within Gly-X-Y repeats, forming the collagen triple helix within the collagenous domain, is often considered for *COL7A1* reframing strategies [35,36,37].

Chamorro et al. [35] described the first exon reframing approach in genodermatoses. This ex vivo approach involved adenoviral delivery of a TALEN pair, targeting a pathogenic cytosine insertion in exon 80 of *COL7A1*, directly into immortalized patient keratinocytes. Over 70% of modified keratinocyte clones demonstrated C7 protein re-expression, with the majority of indels comprising reframing 1 bp deletions. Subsequent engraftment of reframed samples confirmed efficient phenotypic restoration, although expression and C7 localization appeared divergent between distinct indels. Due to the success of the reframing strategy developed by Chamorro et al. [35], the approach was implemented in primary patient keratinocytes [36]. Modification efficiencies in primary patient cell-derived keratinocyte clones ranged from 9% to 11%. The previously described tendency for indels comprising reframing 1 bp deletions was observed, with 75% of modified clones demonstrating C7 re-expression. To confirm functionality of reframed *COL7A1*, expanded clonal samples were engrafted onto mouse models. Subsequent analysis indicated abnormal localization of variant C7 and reduced mechanical resistance in associated grafts. This was suggested to be a result of four miscoded amino acids, including two glycine residues, following introduction of 1 bp deletions at the TALEN target site [36].

Recently, Takashima et al. [37] described an ex vivo reframing approach, targeting RDEB patient fibroblasts. A single mutation-specific CRISPR/Cas9, targeting a cytosine deletion within exon 70 of *COL7A1*, was delivered via plasmid into RDEB fibroblasts prior to selection of transfected cells. In immortalized fibroblasts, targeting efficiencies of 56% were achieved, with over 60% of these leading to reframing. This targeting strategy resulted in one to three miscoded amino acids following reframing, with many outcomes not altering critical glycine residues. Subsequent triple-helix formation analysis of samples, harboring the two most frequent reframing indels, indicated correct functioning of reframed C7. These samples also demonstrated transcription, translation and correct distribution of C7 from reframed alleles. The efficiency of this approach was confirmed following similar treatment and analysis of primary patient fibroblasts. Fluorescence-activated cell sorting (FACS) analysis indicated C7 expression in approximately half of treated cells and injection of bulk-treated samples into a mouse model led to C7 rescue of the fragile basement membrane zone (BMZ) [37].

Exon reframing approaches hold great promise for the correction of many severe recessive disorders. As with gene disruption approaches, the advent of precise CRISPR/Cas9 targeting [62], appears to underline this further. Targeting of pathogenic indels with nucleases that generate highly homogenous DSB repair outcomes could lead to highly efficient approaches with minimal amino acid divergence. This could enable the use of bulk-genome edited samples in therapeutic applications, precluding the need for single cell expansion and screening. Additionally, in vivo application of highly precise molecules is likely feasible, although the immunogenicity of the still heterogeneous reframed proteins would require further analysis. However, the requirement for minimal amino acid divergence from the wild-type would likely necessitate costly mutation-specific genome editing and approach-specific functional analysis.

The promising potential for precise exon reframing suggests that additional genodermatoses targets should be considered. These would likely include other dermatological genes that frequently feature recessive mutations, such as other collagens, laminins and integrins. The keratins are unlikely to represent strong candidates, due to their strong tendency for dominant-negative mutations and fragility with mild amino acid code variance. However, precise strategies targeting frameshift-associated keratin tail mutations, such as those responsible for congenital reticular ichthyosiform erythroderma [67] could hold promise.

Still, the functionality of reframed proteins must be accounted for in the design of future approaches. This could potentially involve analysis of reframing nuclease activity 5′ and 3′ of pathogenic indels, with proximity to the mutation representing a critical consideration.

### 2.3. Exon Deletion

Excision of pathogenic exonic sequences is a proven strategy for the partial correction of several genetic disorders, such as Duchenne muscular dystrophy [68,69,70,71,72]. Previously, genodermatoses-related therapeutic approaches based upon exon ‘skipping’ were successfully achieved at the RNA level [66]. However, exon deletion strategies have recently been implemented in several dermatological genome editing therapy approaches [38,39].

The nature of CRISPR/Cas9 sgRNA complexing enables the targeting of distinct genomic loci in tandem [12]. As a result, dual CRISPR/Cas9 targeting can be used for highly efficient excision of the intervening sequence [39]. Efficient exon deletion approaches implement CRISPR/Cas9 targeting of introns 5′ and 3′ of the target exon [38,39]. The distal DSB ends are subsequently repaired via classical EJ and alternative EJ repair pathways [15,25,73], frequently leading to highly efficient, homogenous indel outcomes. This is particularly true on the RNA level [39]. However, safety concerns persist with this approach, relating to the generation of large (several kb) on-target deletions [15,25,73]. Furthermore, the use of dual CRISPR/Cas9 targeting strategies is likely to lead to potential doubling of nuclease off-target sites.

Exon deletion approaches can theoretically be implemented for the correction of dominant and recessive disease forms. This approach can be employed to effectively excise any mutation type from mutant alleles, while maintaining or restoring the wild-type reading frame. However, reading frame maintenance necessitates the deletion of an in-frame exon. Additionally, not all genes or exons are suitable for exon deletion, as resulting structural alterations might affect transcript or protein functionality. Each exon deletion approach therefore requires full characterization, although genes and exons encoding amino acid repeats appear amenable to internal truncation [36,38,39,63,68,69,72,74]. Exon deletion can additionally provide an effective means of gene knock-out, if the resultant deletion alters the reading frame.

Within genodermatoses, all exon deletion approaches to date have been performed on *COL7A1* [38,39]. The *COL7A1* gene is particularly suitable for exon deletion approaches, as the triple helix-encoding region is comprised of short exons encoding G-X-Y amino acid repeats. Additionally, these exons are in-frame and feature intact codons [75]. Truncated C7 variants lacking sequences encoded by specific collagenous domain exons 70 [74], 73, 80 [76] and 105 [77] have been shown to retain functions of the full-length proteins.

Although over 650 different RDEB-associated mutations within *COL7A1* have been described [78,79], exon 80 represents the target of all gene deletion approaches in genodermatoses to date [38,39]. This is largely due to the frequency of several highly prevalent mutations within this region of the gene. Indeed, a single c.6527insC mutation represents 46% of RDEB alleles in Spanish populations [80] and has been proposed as the most prevalent *COL7A1* mutation [39]. An exon 80-specific exon deletion strategy might therefore provide benefit to a large proportion of genodermatoses patients.

Wu et al. [38] described the first gene deletion approach in genodermatoses. This strategy involved the in vivo delivery of dual CRISPR/Cas9 ribonucleoproteins (RNPs) into an RDEB mouse model via intradermal injection into the tail skin and subsequent electroporation. Cas9-mediated excision of exon 80 was subsequently confirmed at the DNA and RNA level. Although the authors noted that only 2% of epidermal cells had been targeted by the procedure, enrichment of BMZ-localized C7 and increased stability of the DEJ was observed. However, long-term phenotypic correction was not described as analysis was performed 3-5 days post-treatment [38].

Bonafont et al. [39] recently described a similar dual CRISPR/Cas9 RNP electroporation strategy in primary human keratinocytes, proposing an ex vivo therapeutic approach. This followed prior observations that single nuclease-mediated deletion of exon 80 in an RDEB patient keratinocyte clone restored C7 expression and resulted in phenotypic correction [36]. Bonafont et al. [39] achieved auspicious exon deletion efficiencies of over 80% in bulk-treated primary keratinocytes. Restored C7 transcription and translation were subsequently confirmed prior to the long-term engraftment of bulk-treated samples, whereby highly efficient phenotypic restoration was confirmed.

Exon deletion appears to represent a highly promising approach to therapeutic gene editing. It theoretically enables the development of a single genome editing approach to treat multiple mutant alleles in non-patient specific manners, unlike many other strategies. This appears particularly relevant for mutation hotspots, such as exon 80 of *COL7A1*. Furthermore, the typically efficient and uniform indel outcome, at the RNA and protein level, facilitates the potential implementation of this strategy in vivo or ex vivo via application of bulk-treated samples. However, future considerations for this strategy might center upon the potential introduction or ablation of splice sites within targeted intronic sequences [39].

The auspicious editing and correction efficiencies achieved for *COL7A1* exon 80 deletion suggest that this approach could be expanded to additional exonic targets. These could comprise exons 29–112, encoding the C7 collagenous domain and harboring a large proportion of described nonsense mutations [39]. Furthermore, genodermatoses-associated genes featuring long, repetitive coding sequences, such as additional collagen genes, might represent promising exon deletion targets for future studies and therapies. However, the preservation of open reading frames and amino acid-encoding repeats must be accounted for in the design of future approaches.

### 2.4. Exon/Gene Insertion

The possibility of directly correcting several mutations with edits of various sizes makes HR a very attractive alternative to EJ-based strategies, applicable both in autosomal dominant and autosomal recessive diseases. Exon/gene insertion approaches utilize HR DSB repair machinery, requiring a repair template that harbors left and right homology arms (HAs) for precise insertion of large DNA fragments [81]. However, targeted integration of transgenes is generally inefficient, largely as HR is active only during the late S/G2 phase and must compete with EJ-based repair pathways [81].

Currently, all attempts to correct mutations within genodermatoses via HR-mediated insertions are dependent on selection due to their very limited efficiency [35,41,42,43,44,45]. The majority of insertion approaches reported to date have been performed in RDEB, targeting *COL7A1*.

As a first proof of concept for precise gene addition, Coluccio et al. [40] targeted a “safe harbor” locus, the adeno-associated virus integration site 1 (*AAVS1*), by ZFN-induced HR in a human keratinocyte cell line and in primary keratinocytes. Their data clearly indicated poor induction of the HR-dependent DNA repair pathways, especially in primary keratinocytes with <1% GFP integration, a significant limitation of targeted gene integration [40]. These relatively low HR efficiencies suggested use of selection-based systems and expansion of corrected single-cells clones for future studies. Sebastiano et al. [42] demonstrated nuclease-free targeting of mutations in exons 2 and 3 of the *COL7A1* locus. This was achieved using an AAV-mediated system that spanned six exons and contained 1.4 kb targeting arms on either side of a central puromycin selection cassette. The authors compared the targeting efficiency of nuclease targeting (up to 11%) with that induced by the novel adeno-associated viral variant (AAV-DJ) (up to 57%), identified as having a high recombinogenic activity. Single cell clone analysis of treated iPSCs revealed up to 100% correctly targeted clones [42].

TALEN-mediated gene insertion was first demonstrated in patient RDEB fibroblasts carrying 1837C > T PTC mutation by Osborn et al. [41]. Correction was achieved by non-viral TALEN delivery as mRNA or plasmid DNA together with a plasmid DNA template flanking exon 12–15. Within the donor plasmid was a floxed-phosphoglycerate kinase promoter (PGK)-puromycin cassette oriented in the way that it would be inserted into the intron between exons 12 and 13, thus allowing for Cre-recombinase–mediated removal. Gene-edited fibroblasts were reprogrammed into iPSCs and tested in an in vivo model for their capacity to deposit C7 at the DEJ [41]. Chamorro et al. [35] compared correction efficiencies of an EJ-based reframing, with an HR-based strategy. For HR they combined AAV-mediated delivery of donor template DNA with TALENs expressed by adenoviral vectors to address the correction of the c.6527insC mutation in the *COL7A1* gene. They designed AAV vector-based targeting constructs including a wild-type exon 80 in the right donor arm, and neomycin resistance (neo)-expressing cassettes flanked by AAV-packaging signals. The authors could achieve targeting efficiencies of up to 39% in immortalized RDEB keratinocytes with 94% of recombined clones carrying the corrected allele. Interestingly, when comparing their two approaches, they could see that C7 restoration in clones corrected by HR was in the range of control keratinocytes, whereas in contrast to it, several EJ-reframed clones showed C7 overexpression [35]. A very similar strategy for HR-mediated correction of two distinct frameshift mutations in the *COL7A1* gene was applied by Webber et al. [43] and Hainzl et al. [44], thereby introducing a puromycin selection cassette into an adjacent intron for selection and subsequent Cre-recombinase-mediated removal. Both groups used non-viral-based ex vivo HR strategies with CRISPR/Cas9 nuclease and nickase. Hainzl et al. [44] co-transfected RDEB keratinocytes with the donor DNA template (plasmid), carrying mRuby/Puromycin to facilitate clonal selection, and CRISPR/Cas9 reagents targeting 6527insC mutation within exon 80. Subcloning analysis of selected bulk populations revealed 17% corrected alleles for SpCas9 and 24% for SpCas9 D10A [44]. In contrast, Webber et al. [43] designed a double-stranded DNA donor template and electroporated it with either nuclease or nickase version of Cas9 and sgRNA into fibroblasts that were then puromycin-selected in bulk. Both analyzed bulk populations showed HR at genomic level to some extent. Importantly, the SpCas9 D10A treated sample did not show any mutagenic EJ-events at the on-target locus and therefore, nickase gene-corrected cell clones were selected for reprogramming into iPSCs and successfully differentiated into keratinocytes in vitro expressing keratinocyte stem cell markers [43]. A novel strategy for the in situ correction of JEB mutations via HR-mediated repair was successfully achieved in patient keratinocytes carrying a homozygous frameshift mutation in exon 14 in the *LAMB3* gene. Benati et al. [45] described a strategy to insert a complementary DNA (cDNA) cassette containing *LAMB3* exon 3 to the end of the gene, flanked by a splice acceptor into the endogenous locus of the gene such that it is regulated by its own natural regulatory elements. They packaged an adenovector carrying Cas9/sgRNA tailored to the intron 2 of *LAMB3* gene and an integration defective lentiviral vector bearing the repair template to apply CRISPR-mediated HR to safely in situ integrate a therapeutic *LAMB3* cDNA in JEB keratinocytes. Barely detectable 0.48% correct HR efficiency made positive selection mandatory [45]. Osborn et al. [46] employed a different strategy, aiming for distinct cell types as a source of missing C7 expression in RDEB. For their strategy a sgRNA targeting 164 bp upstream of the *COL7A1* start codon and a donor template carrying a transcriptional promoting element termed UMET, flanked by donor arms spanning the *COL7A1* transcriptional start site, were designed. To up-regulate C7 expression an HR-mediated insertion of a transcriptional element upstream the start codon in umbilical cord hematopoietic stem cells (HSCs) and peripheral blood T cells was performed. Therefore, the donor template was packaged into AAV-6 serotype and cluster of differentiation 34 (CD34) + CD133 + HSCs were infected along with Cas9 RNP complexes electroporated. This efficient gene targeting and HR inducing method (>60%) allowed high C7 levels in HSCs and T-cells [46].

Still, the majority of studies showed, that HR rates can be modest and the DSBs that initiate HR commonly result in accompanying undesired indels, unless using the SpCas9 D10A version. HR can be used to modify any genomic sequence from a donor template [41]. However, the efficiency in therapeutically relevant cells is typically very low, often necessitating antibiotic resistance cassettes to enrich for corrected cells [41,43,44]. Since DSB repair typically results in an excess of EJ generated indels accompanying the desired HR product, the ability to achieve robust allele correction with high efficiency and minimal byproducts (e.g., indels from EJ) is often critical. Different gene editing approaches for correction of the EB phenotype have been described so far, but they either suffer from low correction efficiencies or rely on the deletion of exonic sequences [36,37,39,41,43,44,45,48,49,50]. Owing to low editing efficiency, the majority of the above-mentioned studies included a selection step of cells in bulk, limited dilution, clonal selection, single-cell expansion, and screening of individual clones. For most antibiotic selections, a selection cassette flanked by loxP sites was employed that, after Cre-mediated removal, leaves a loxP footprint in the targeted intron [35,42,43,44]. Still, besides its limitations, HR-mediated insertion appears to represent a highly promising approach, which theoretically enables the development of single non-personalized approaches to treat patients with several distinct mutations in genes, as shown for *LAMB3* [45]. Nevertheless, the typically inefficient and not uniform repair outcome facilitates the potential implementation of this strategy only for ex vivo approaches via application of selected bulk-treated samples or corrected and expanded single-cells clones.

The development of the mutant Cas9 D10A version inducing a single-strand break increases HR compared to EJ and may reduce unwanted off-target effects and toxicity [82,83,84]. However, due to the very low HR inducing efficacy of this mutant Cas9 version makes antibiotic selection or single cell dilutions mandatory for such gene editing approaches [43,44]. An improvement of knock-in efficiency might be possible with the usage of alternative EJ-based methods, which was shown to yield a higher knock-in efficiency, greater than reached with HR- and classical EJ-based strategies. Further, additional DNA cleavage on an HR donor can improve the efficiency of homology-mediated gene integration. With this strategy, targeted integration can be achieved via the HR pathway as well as through classical and alternative EJ, thus improving knock-in efficiency [81,85,86]. EJ-based strategies can therefore introduce a targeted integration in a homology-dependent manner, making DNA segment replacement in the genome practicable, compared to classical EJ-based targeted integration, where only introduction of a donor DNA segment into the cutting site is possile, making it unsuitable for replacing a mutated sequence (such as a point mutation) with the correct one [81]. The use of nickase-based strategies should be considered for future precise gene integration approaches, due to the lower risk for unwanted off-target effects and increased HR efficiencies. A prerequisite for effective HR-mediated correction of a mutation is the inactivation of the respective protospacer adjacent motif (PAM) sequence located on the repair template to avoid further cutting of already repaired DNA sequences. Template cutting or nicking of the repair template could further improve repair efficiencies via taking advantage of the above-mentioned alternative EJ pathway in addition to the HR pathway. In general, all strategies should have the cutting site as close to the mutation as possible to increase correction efficiencies. In addition, and especially true for compound heterozygous mutations, allele specificity would be a further wanted improvement of such approaches.

The promising potential for precise insertions of repair templates can be theoretically used to correct any kind of mutation and every gene causing distinct genodermatoses. These would likely include other dermatological genes that frequently feature recessive as well as dominant mutations, such as the collagens, laminins, integrins and keratins. One obvious disadvantage of this approach is the introduction of small exogenous and foreign DNA fragments in the human genome and it can therefore not be considered as traceless. Another significant concern is that these selection-based strategies may lead to targeted or random integration of the entire donor plasmid or large parts.

### 2.5. Homologous Recombination

The main aim of HR-based approaches without insertions of whole exons or genes is the traceless correction of genes harboring any kind of mutation. With HR-mediated targeting strategies, it is possible to directly revert a disease-causing variant in a gene via a single-nucleotide change. In contrast to the section above, HR-based strategies are only efficient in a patient personalized manner. A repair template, including a longer homology wild-type sequence and therefore covering more mutations sites, would result in several different not predictable HR events, which would even lower the already limited HR efficiency. So far, such personalized gene editing approaches via HR have been performed in cells isolated from XP, EBS and RDEB patients showing the potential to correct autosomal dominant as well as autosomal recessive diseases [47,48,49,50,51].

In 2013 Dupuy et al. [47] used engineered MN and TALE nuclease to promote the targeted correction of *XPC* mutation in the XP4PA cell line, derived from fibroblasts of a Xeroderma pigmentosum group C (XP-C) patient, which carries the homozygous autosomal recessive DTG mutation in the *XPC* gene. They showed, that both MN- and TALEN-assisted targeted approaches allowed successful correction (2.5% correction) of the *XPC* founder mutation in an XP4PA cell line of XP-C patients without selection marker. Because *XPC* is an autosomal recessive disease, they postulated that a monoallelic correction of only few keratinocytes might be sufficient for clinical application [47]. Another MN-based HR approach was used in 2016 to correct two mutations identified in patients with RDEB [48]. Similar to Coluccio et al. [40] integration-deficient lentiviral vectors were used to deliver MNs and Donor. They targeted intron 2 of *COL7A1* in immortalized keratinocytes, primary keratinocytes and primary fibroblasts with targeting efficiencies of 9%, 7.5% and 2.2% respectively and reached ~4% C7 restoration. Despite *COL7A1* correction in unselected primary keratinocytes and fibroblasts, the frequency of DSB-mediated HR was still too low to consider MNs for ex vivo applications [48]. In 2018, the same group demonstrated efficient *COL7A1* editing in primary RDEB keratinocytes and RDEB fibroblasts based on CRISPR/Cas9, without any antibiotic- or fluorescence- based selection [50]. They demonstrated functional rescue of type VII collagen expression in primary RDEB fibroblasts and keratinocytes, and anchoring fibril (AF) formation in an ex vivo xenograft model using gene-edited RDEB skin grafts. The delivery of integration-defective lentiviral vectors (IDLVs) with the specific sgRNA together with Cas9 nuclease and the donor template in RDEB cells resulted in 11% C7 restoration, which increased up to 20–26% of C7 expression in a continuous linear staining pattern along the dermal–epidermal junction following transplantation of gene corrected skin equivalents. Importantly, these results are in favor of local diffusion of C7 at the sites of synthesis and its accumulation at the dermal–epidermal junction [50]. Still, relatively low HR efficiencies accompanied by high risks of off-target effects [87] have impeded CRISPR/Cas9′s way into the clinic as a curative treatment for monogenetic disorders. Ran et al. [84] described a method to increase HR rates and to reduce the frequency of off-target events by using the Cas9 mutant D10A, which preferably induces single-strand breaks (nicks) within the DNA, in a double-nicking configuration. While the usage of the D10A Cas9 nickase is known to facilitate HR at the on-target region, the combined application of two nickases in a double-nicking configuration further improved its efficiency, which is, in single-nicking approaches, substantially reduced in comparison with the wild-type Cas9. Kocher et al. [49] described a CRISPR/D10ACas9-mediated double-nicking strategy for the specific repair of a dominant mutation within exon 6 of the *KRT14* gene, which causes generalized severe EB. Co-delivery of a Cas9 D10A nickase, a predicted sgRNA pair specific for intron 7, and a minicircle donor vector harboring the homology donor template into EBS keratinocytes resulted in a recombination efficiency of >30% and correction efficiency of 16% of the mutant *KRT14* allele after antibiotic selection [49]. In 2019 Kocher et al. [51] presented a very similar selection-based double-nicking strategy for HR-mediated correction of a splice-site mutation in exon 3 (c.425A > G) of *COL7A1*. Extensive comparison of HR efficiencies mediated by either Cas9, single-nicking, or double-nicking highlighted, that double-nicking consistently outperformed all other approaches. They accomplished remarkably high HR frequencies of 89% and C7 restoration of 77% with double-nicking while at the same time keeping unwanted repair outcomes, such as EJ, at a minimum (11%). Interestingly, subtle template modifications and strategic nicking of templates could further improve HR efficiencies in treated RDEB patient keratinocytes [51]. Taken together, all these works provide a framework for efficient, precise, and safe repair of genes, such as *COL7A1*, which lies at the heart of any future curative therapy of genodermatoses. Still, relatively low HR efficiencies accompanied by high risks of off-target effects and very efficient selection-based strategies with the possibility of targeted or random integration of the entire donor plasmid or large parts thereof, have impeded CRISPR/Cas9′s way into the clinic. Clearly, improved HR efficiencies and predictable repair outcomes are pivotal for advancing experimental gene therapies into clinical settings.

Numerous aspects have an influence on HR efficiencies, including delivery and modus operandi of DNA-modifying agents, the nature of the repair template, or cell type and cell cycle stage. High efficiency, high precision, and seamless repair all need to be carefully balanced in future ex vivo therapies to circumvent the need for antibiotic selection and clonal expansion of corrected cells. Although Cas9 is highly efficient in inducing DNA DSBs and ensuing DNA repair, subtle modifications in experimental design can significantly alter the outcome of such efforts [51]. PAM inactivation on the donor template, the close proximity of the cutting site to the mutation, paired-nicking and additional nicking of the repair template seem to increase HR efficiencies. Going forward, we believe that combining RNP delivery, which has been shown to provide increased targeting efficiency compared to plasmid transfection [39], higher rates of editing and shorter persistence in the nucleus, with double-nicking and optimized repair templates will offer the best option for correction of gene mutations in terms of efficiency and precision.

Especially for recessive missense mutations, HR approaches should be the first choice for precise correction of the respective mutation.

## 3. Future Prospects of Gene Editing in Genodermatoses

Several recent CRISPR/Cas9 developments will likely lead to rapid advances in the near future, potentially propelling the application of this technology into the clinic. Delivery of CRISPR/Cas9 into target cells via electroporation of RNPs has been shown to result in rapid, high on-target efficiencies and low off-target activity [39]. Furthermore, Good Manufacturing Practice (GMP)-grade Cas9 protein and sgRNA are available. Protein-based delivery of Cas9 circumvents potentially detrimental integration of commonly used DNA-based delivery vectors. Further, several studies have recently revealed more precise, predictable outcomes, following targeting via a single CRISPR/Cas9 nuclease, at many loci [88,89]. As these models are primarily based upon the sequence context of the PAM site, they enable rapid and efficient development of precise CRISPR/Cas9-based strategies resulting in predictably homogenous DSB repair outcomes for reframing and knockout approaches. Specific DSB repair outcomes might also be preferentially selected through precise modulation of the cell cycle. However, clinical application of such modulation would likely require compound-free stimulation of or stalling in specific cell cycle phases.

Another promising avenue of development in the field of genome editing has emerged with the advent of base editing. CRISPR/Cas9 base editors can be used to target site-specific base deamination-induced base transition without DSB generation [90,91,92]. Currently, two types of base editors have been developed, both chimeric fusion proteins utilizing the sgRNA-targeting and target strand-nicking activity of Cas9 D10A nickase. Adenine base editors utilize both a wild-type TadA adenosine deaminase and an evolved TadA * deoxyadenosine deaminase to deaminate adenine bases on the non-target strand. This enables A•T→G•C and T•A→C•G base editing, within a small region of DNA displaced by sgRNA binding, depending upon PAM and target orientation [92]. In principle, cytosine base editors (CBEs) work in a similar manner, utilizing rAPOBEC1 to deaminate cytosine on the non-target strand. This enables C•G→T•A and G•C→A•T base editing [90].

This technology therefore has potential applications in gene disruption, circumventing the need for DSB and PTC induction via direct PTC generation. Genome editing-associated instability should therefore be much reduced compared to established gene disruption strategies. This should also lead to a reduction of heterogeneous on-target indels, typically associated with gene disruption approaches, although low frequency nickase-associated indels have been described [93]. However, prior characterization of target PTCs is still recommended to avoid the generation of truncated protein variants. Base editing also provides a much more efficient method of precisely correcting missense or nonsense mutations, frequently the cause of many genodermatoses, in comparison to established HR-based approaches. Additionally, as exogenous DNA templates are not required, potentially detrimental vector integrations should be reduced [94].

Recently, Osborn et al. [52] described the first base editing approach in genodermatoses. This ex vivo approach involved targeting of two distinct *COL7A1* mutations with ABEs. ABEs were co-delivered with sgRNAs as mRNA via electroporation into RDEB patient fibroblasts and iPSCs. In primary fibroblasts, correction rates of over 20% and over 40% were achieved at the DNA and RNA level respectively. This resulted in restoration of full-length C7 expression that demonstrated correct deposition at the BMZ in a murine teratoma model [52].

However, several considerable disadvantages with base editing technology persist. Primarily, the non-specific nature of base editing within the region of DNA displaced by the sgRNA necessitates efficient strategy design and precludes the use of this technology in repetitive target sites. Off-target activity was recently demonstrated in human fibroblasts [52]. Furthermore, both CBEs and ABEs appear to induce edits within RNA, which can be considered off-targets. These have been shown to occur on a transcriptome-wide level, in both protein-coding and non-protein-coding sequences [95]. However, continued improvements of the technology appear to promise a reduction in these potentially detrimental events [95].

Despite these improvements, several valuable genome editing-mediated approaches cannot be achieved with base editing, such as exon deletion, exon/gene insertion and transversion. The recently described prime editing may enable highly efficient introduction of these outcomes without the generation of DSBs, accompanied with reduced off-target activity [96]. However, the clinical applicability of this technology remains to be seen.

Although the accessible nature of the skin theoretically enables careful monitoring of edited tissues in genodermatoses patients, emerging considerations about the therapeutic application of genome editing largely center on safety concerns. Issues with off-target activity can be mitigated through the use of improved Cas9 variants and sgRNA design [49,51,84,97]. However, a defined level of safe off-target indels remains elusive, with a lack of data-driven guidance [98]. Additionally, several recent studies have indicated increased genome editing efficiencies in cells with reduced activation of the P53 pathway [99]. Moreover, increased activation of this pathway is associated with prolonged nuclease activity [100]. While this indicates that genome editing triggers cell cycle arrest and apoptosis [99,100], it could further suggest that surviving, proliferating genome-edited cells might display pro-tumorigenic capabilities. Other recent studies have described frequent large on-target deletions [73,101] and P53-dependant chromosomal translocations associated with genome editing [102]. This may have remained uncharacterized until recently due to PCR- and sequencing-based approaches involving over-amplifications of shorter genomic regions. The failure to detect and analyze such gross chromosomal aberrations, represents a severe oversight in the development of genome editing therapies, some of which are currently in clinical trial phases. Furthermore, it is likely that the large deletions observed at on-target sites occur at difficult-to-analyze off-target locations.

Despite emerging safety concerns, genome editing appears to represent the ultimate solution for curing genetic disorders. However, for clinical application of genome editing further considerations may prove necessary—homogenous genome editing outcomes, minimally at the RNA level, and non-patient specificity [39]. These considerations are likely to make genome editing approaches attractive to pharmaceutical partners that require a single, predictable compound for clinical trial and marketing.

## Figures and Tables

**Table 1:** Genome editing approaches for genodermatoses.

Targeting Strategy (Nuclease)	Gene Target (Mutation)	Mutation Type	Dominant vs. Recessive	Disease	Ex Vivo vs. In Vivo	Pub.
Gene Knockout (TALEN, CRISPR/Cas9)	*COL7A1* (c.8068_8084delinsGA)	Non-Frameshift Indel	Dominant	DDEB	Ex Vivo	[31]
Gene Knockout (TALEN)	*KRT5* (c.556G > T, c.1424A > G)	Missense, Missense	Dominant, Dominant	EBS	Ex Vivo	[32]
Gene Knockout (CRISPR/Cas9)	*KRT9* (c.434delAinsGGCT)	Non-Frameshift Indel	Dominant	EPPK	In Vivo	[33]
Gene Knockout (TALEN)	*KRT10* (c.1333G > A, c.481_486delTTGGAC)	Non-Frameshift Indel, Missense	Dominant, Dominant	EI	Ex Vivo	[34]
Gene Reframing (TALEN)	*COL7A1* (c.6527insC)	Frameshift Indel	Recessive	RDEB	Ex Vivo	[35]
Gene Reframing (TALEN)	*COL7A1* (c.6527insC)	Frameshift Indel	Recessive	RDEB	Ex Vivo	[36]
Gene Reframing (CRISPR/Cas9)	*COL7A1* (c.5819delC)	Frameshift Indel	Recessive	RDEB	Ex Vivo	[37]
Exon Deletion (Dual CRISPR/Cas9)	*COL7A1* (c.6485G > A)	Nonsense	Recessive	RDEB	In Vivo	[38]
Exon Deletion (Dual CRISPR/Cas9)	*COL7A1* (c.6527insC)	Frameshift Indel	Recessive	RDEB	Ex Vivo	[39]
Exon/Gene Insertion (ZFN)	*AAVS1* (N/A)	N/A	N/A	N/A	Ex Vivo	[40]
Exon/Gene Insertion (TALEN)	*COL7A1* (c.1837C > T)	Nonsense	Recessive	RDEB	Ex Vivo	[41]
Exon/Gene Insertion (ZFN, TALEN, CRISPR/Cas9)	*COL7A1* (c.356_357delCA, c.90delC)	Frameshift Indel Frameshift Indel	Recessive	RDEB	Ex Vivo	[42]
Exon/Gene Insertion (TALEN)	*COL7A1* (c.6527insC)	Frameshift Indel	Recessive	RDEB	Ex Vivo	[35]
Exon/Gene Insertion (CRISPR/Cas9)	*COL7A1* (c.4317delC)	Frameshift Indel	Recessive	RDEB	Ex Vivo	[43]
Exon/Gene Insertion (CRISPR/Cas9)	*COL7A1* (c.6527insC)	Frameshift Indel	Recessive	RDEB	Ex Vivo	[44]
Exon/Gene Insertion (CRISPR/Cas9)	*LAMB3* (c.1945dupG, c.1903C > T)	Frameshift Indel Nonsense	Recessive	JEB	Ex Vivo	[45]
Exon/Gene Insertion (CRISPR/Cas9)	*AAVS1* (N/A)	N/A	Recessive	RDEB	Ex Vivo	[46]
Homologous Recombination (Meganuclease, TALEN)	*XPC* (c.1643_1644delTG)	Frameshift Indel	Recessive	XP	Ex Vivo	[47]
Homologous Recombination (Meganuclease)	*COL7A1* (c.189delG, c.425A > G)	Frameshift Indel Splice Site Mut.	Recessive	RDEB	Ex Vivo	[48]
Homologous Recombination (Dual CRISPR/Cas9 D10A nicking)	*KRT14* (c.1231G > A)	Missense	Dominant	EBS	Ex Vivo	[49]
Homologous Recombination (CRISPR/Cas9)	*COL7A1* (c.6527insC)	Frameshift Indel	Recessive	RDEB	Ex Vivo	[44]
Homologous Recombination (CRISPR/Cas9)	*COL7A1* (c.189delG)	Frameshift Indel	Recessive	RDEB	Ex Vivo	[50]
Homologous Recombination (Dual CRISPR/Cas9 D10A nicking)	*COL7A1* (c.425A > G)	Splice Site Mut.	Recessive	RDEB	Ex Vivo	[51]
Base Editing (ABE)	*COL7A1* (c.553C > T, c.1573C > T)	Nonsense	Recessive, Recessive	RDEB	Ex Vivo	[52]

*Abbreviations:* CRISPR (clustered regularly interspaced short palindromic repeats)/Cas9 (CRISPR associated protein 9), TALEN (transcription activator-like effector nuclease), ZNF (zinc-finger nuclease), RDEB (recessive dystrophic epidermolysis bullosa), DDEB (dominant dystrophic epidermolysis bullosa), JEB (junctional epidermolysis bullosa), EBS (epidermolysis bullosa simplex), EP (xeroderma pigmentosum), EI (epidermolytic ichthyosis), EPPK (epidermolytic palmoplantar keratoderma).
